# Analyzing SDG interlinkages: identifying trade-offs and synergies for a responsible innovation

**DOI:** 10.1007/s11625-023-01336-x

**Published:** 2023-05-26

**Authors:** Nikki Luttikhuis, Kirsten S. Wiebe

**Affiliations:** 1 Sustainable Energy Technology, SINTEF, Torgarden, P.O. Box 4760, 7465 Trondheim, Norway; 2 Department of Industrial Economics and Technology Management, Norwegian University of Science and Technology (NTNU), 7491 Trondheim, Norway

**Keywords:** Sustainable Development Goals, Agenda 2030, RI, Point of care

## Abstract

This paper responds to recent calls to address the indivisible nature of the Sustainable Development Goal (SDG) framework and the related knowledge gap on how SDG targets interlink with each other. It examines how SDG targets interact in the context of a specific technology, point of care (PoC) microfluidics, and how this relates to the concept of responsible innovation (RI). The novel SDG interlinkages methodology developed here involves several steps to filter the relevant interlinkages and a focus group of experts for discussing these interlinkages. The main findings indicate that several social synergies occur when deploying PoC microfluidics, but that the environmental trade-offs may jeopardize the total progress toward the SDGs. More specifically, the environmental sacrifices (use of plastics and lack of recyclability) resulted in the product being cheaper and, thus, better accessible. This work suggests that attention should be given (and prioritized) to the use of renewable and recyclable materials without jeopardizing the accessibility of the product. This should minimize the identified trade-offs. These findings inform how analyzing SDG interlinkages relates to the responsibilities and dimensions of RI in several ways. First, analyzing SDG interlinkages helps to execute the governance responsibility by using the RI dimensions (anticipation, reflexivity, inclusion and responsiveness). Second, analyzing SDG interlinkages gives insights into if and how a technology relates to the do-good and avoid-harm responsibility. This is important to assess the responsiveness of the technology to ensure that the technology can become truly sustainable and leaves no one behind.

## Introduction

The Sustainable Development Goals (SDGs), as part of the 2030 Agenda for Sustainable Development, are designed to be treated as universal and indivisible. Universality relates to the fact that the SDGs apply to all nations, from low- and middle-income countries to the highest-income countries. Indivisibility means that the SDGs are part of an integrated framework and should be addressed as such (Bennich et al. 2020). Recently, an increasing amount of papers have been targeting the indivisible nature of the SDG framework by looking at interlinkages for policy coherence (McGowan et al. 2019). Previous scholars that have researched this topic emphasized that analyzing SDG interlinkages is always context specific and cannot easily be generalized, be it a country, region or technology (Nilsson et al. 2016; Weitz et al. 2018). This paper also addresses this indivisible character of the SDG framework and focuses on the challenge to identify how the SDG targets interlink from a technology perspective.

Technologies and innovations play an important role in achieving the SDGs. To achieve the SDGs, it is essential that these technologies are produced and consumed responsibly, mostly reflected in SDG 12 *Responsible Consumption and Production*. Currently, however, increasing welfare and economic growth are coupled with larger environmental and material footprints, especially in developed countries (Pradhan et al. 2017), hampering progress toward SDG 12. Pradhan et al. (2017) found that SDG 12 is associated with the most trade-offs from other goals, underlining the importance that technologies need to be both consumed and produced responsibly to address all SDGs (Voegtlin and Scherer 2017). For this purpose, scholars have stressed three responsibilities that are important to consider when defining a responsible innovation (RI): the avoid-harm, do-good and governance responsibilities (Stahl and Sully de Luque 2014; Voegtlin and Scherer 2017). Previous research within the area of responsible innovation (RI) looked at how this concept links toward the SDGs. Nylund et al. (2021) mentioned that the introduction of the SDGs offered a consensus in defining what exactly constitutes a responsible innovation. This forced corporations to indulge in RI and show their impacts on society and the environment, especially, the detailed SDG targets have been regarded as important tools to strategically align RI activities with these targets (Nylund et al. 2022). Thus, the clarity of the SDG targets is helpful to break down, improve and measure RI activities (Nylund et al. 2022; Yaghmaei and Poel 2021), and vice versa, the dimensions and responsibilities of the RI framework help to conceptualize the requirements of innovators to impact and achieve the SDGs (Nylund et al. 2022; Stahl et al. 2019).

How pursuing certain SDG targets may influence progress on other SDG targets has been recognized as a critical knowledge gap within the SDG literature (UN 2016; Weitz et al. 2018). Lately, an increasing number of scholars have tried to fill this knowledge gap and researched interlinkages of SDG targets in detail within a policy context, across different countries (see e.g., Hernández-Orozco et al. 2022 or Weitz et al. 2018). However, how a technology can create interlinkages (i.e., trade-offs and synergies) between the SDG targets remains unexplored. The development of the renewable energy industry is a prominent example of how a technology can create long-term trade-offs: social benefits (more access to affordable energy) comes at an environmental cost (mortality of birds and sea creatures) (Leung and Yang 2012; Odeku 2013). By identifying the (inter)linkages of a technology toward SDG targets, one can create insights into synergies (which will accelerate goal achievement) and trade-offs (which need special attention as they may hamper achievement toward goals). It is critical to understand how movement toward one goal can accelerate or hamper achievement of another goal, as this gives insights into what the exact impact of a technology is (or can be) on achieving the SDGs.

To address the SDG interlinkages in the context of a technology, we use the responsible innovation framework to link the role of technologies to the SDG framework. Previous scholars researching RI have stressed the importance of “anticipating” when assessing a responsible innovation. In addition, the long-term so-called “double effects” of technologies can undermine the avoid-harm and do-good responsibilities associated with RI (Voegtlin and Scherer 2017), which directly relates to the long-term interlinkages between SDG targets. Hence, previous researchers emphasized the relevance of early governance of the positive and negative effects of innovations (Ranabahu 2020; Schomberg 2011; Voegtlin and Scherer 2017). By researching the SDG interlinkages of a technology, we aim to address this anticipatory dimension of the RI concept by providing a long-term focus of the analysis on the 2030 Agenda for Sustainable Development, which the SDGs are part of. This leads to the following research questions:

“How do the targets of the SDGs interlink in the context of a technology?”

“How do the SDG target interlinkages inform the responsibilities and dimensions related to the RI framework?”

The empirical part of this study is executed in the health sector and focuses on point of care (PoC) microfluidic devices, the most well-known example being the rapid antigen COVID-19 test. Ever since the COVID-19 outbreak, we have seen how health technologies that prove to be a game changer in fighting a worldwide pandemic can, at the same time, also create great environmental harm. PoC microfluidic devices have shown to bring healthcare closer to the patient, in a cost-effective way, positively contributing to the social aspect of sustainability. However, these devices do not allow reuse (yet) and are produced from unsustainable materials, creating enormous amounts of waste (Isiksacan et al. 2016; Mejia-Salazar et al. 2020; Trantidou et al. 2018). This sustainability conflict within PoC devices makes this a useful technology to research the SDG interlinkages in depth.

This paper contributes to the literature by identifying how SDG target interlinkages occur in the context of a technological innovation, such as PoC devices. The findings indicate that synergies occur in the socially related targets, but that these synergies are put at risk by the environmental hazards of the technology.

Accordingly, this paper extends the knowledge on how the RI concept links toward the SDG interlinkages and vice versa. It provides an in-depth analysis on SDG target interactions and thus includes the long-term focus of the SDGs into the RI concept. In doing so, analyzing SDG interlinkages plays an important role in the governance responsibility of the RI concept (by using the dimensions of the RI concept) and hence informs the do-good and avoid-harm responsibility. Furthermore, the technology focus of this paper contributes to the SDG interlinkages literature by showing that interlinkages occur in a different manner compared to the vast majority of papers having a policy-oriented focus. It shows that the interlinkages occur from direct strong links to the targets instead of being scattered throughout the entire SDG framework.

## Responsible innovation and SDG interlinkages

Responsible innovation is a framework that evaluates innovations on their potential positive contributions to society (environmentally, economically and socially) on the one hand, and their potential harmful consequences on the other. Lehoux et al. (2018) stressed that RI can enable innovations and technologies to address significant societal challenges, including the SDGs. However, they emphasized that “further reflections and empirical studies are needed since there are deep-seated contradictions” (Lehoux et al. 2018, p. 4) that can limit the potential success of RI and thus progress toward the SDGs. Analyzing SDG interlinkages can support the evaluation of these contradictions of RI.

Stilgoe et al. (2013) have distinguished four dimensions within the RI framework: anticipation, reflexivity, inclusion and responsiveness. These dimensions should be used as an integrated whole. Anticipation involves systematic thinking to increase resilience, while revealing new opportunities. Reflexivity means reflecting on one’s own activities, commitments and assumptions, “being aware of the limits of knowledge and being mindful that a particular framing of an issue may not be universally held” (Stilgoe et al. 2013; Wynne 1993). Inclusion refers to involving stakeholders and members of the wider public in science and innovation practices. Lastly, responsiveness relates to the capacity to change shape or direction in response to stakeholder (and public) values and changing circumstances. Analyzing SDG interlinkages for a technology and/or innovation ensures reflecting and thus anticipating on the long-term interlinked effects on the SDGs, which are set for 2030.

Voegtlin and Scherer (2017) emphasized three responsibilities of innovators when it comes to responsible innovations for sustainable development. The governance responsibility contains the role and responsibilities of businesses in global governance and reflects on the governance structures that should be in place to ensure the necessary regulations and incentives for sustainable practices (Voegtlin and Scherer 2017). This should facilitate the other two responsibilities: do good and avoid harm. Avoiding harm relates to the fact that new products should not have adverse effects on the health of the users, workers and everyone involved; neither should products pollute or harm the environment in any way. Doing good is often reflected in the concepts of social entrepreneurship, eco-innovations or green innovations to create business opportunities. A current limitation in the literature is that these two responsibilities are regarded as two separate aspects that do not necessarily have to be present at the same time, which limits the contribution to sustainable development (Voegtlin and Scherer 2017). On the contrary, both aspects (“avoid harm” and “do good”) should be addressed by innovators to avoid potential trade-offs and to contribute successfully and comprehensively to sustainable development. This directly underlines the importance of analyzing SDG interlinkages, as that can identify how an innovation can create trade-offs and synergies between different SDG targets and thus if and how the technology is doing good or avoiding harm.

Previous scholars have addressed the topic of interlinkages between SDGs in the past, but this research field is diverse in terms of research methods used (quantitative or qualitative), the context in which the interlinkages are researched (countries or policies) and the point of entry (researching a nexus of goals or multiple goals) (Alcamo et al. 2020). Research by Horvath et al. (2022) concluded that the context of the analysis should steer the choice of the method used to analyze SDG interlinkages: the entities analyzed, time and spatial scales, resources and requirements for interdisciplinarity determine the best method fit. This is in line with the argument of Nilsson et al. (2016) and Weitz et al. (2018) who stated that analyzing SDG interlinkages is always context specific and cannot easily be generalized. An overview of the different types of methods, levels of analysis and contexts researched can be found in Appendix 1.

Looking at the research methods used in the SDG interlinkages literature, most of the papers on interlinkages have been of a quantitative nature, using published literature or open-source databases (Bennich et al. 2020). Only a handful of papers have used (semi-)qualitative methods (i.e., qualitative methods with quantitative content analysis) to identify SDG interactions empirically (see e.g., Weitz et al. 2018; Hernández-Orozco et al. 2022). However, many scholars agree that a combination of qualitative and quantitative methods is needed to understand SDG interlinkages, mainly because of data scarcity (see e.g., Pradhan et al. 2017). Most papers have been looking at the country (multiple or single) or region level. For example, Pradhan et al. (2017) looked at interactions within and between SDGs by looking at the correlation of different indicators across 227 countries. Similarly, Anderson et al. (2022) used correlation analysis to identify interlinkages, but used expert knowledge to identify the directional relation. The selection of indicators and methods vary across the quantitative studies performed, leading to different identified interlinkages (Miola and Schiltz 2019; Warchold et al. 2021). Warchold et al. (2022) emphasized that the selection of data is important to consider when assessing interlinkages, as this is one of the reasons the identified interlinkages differ. The study by Castor et al. (2020) is one of the few examples that has not researched SDG interlinkages from a country or region perspective, but instead looked at different energy projects. This is exactly where we also aim to extend the literature: researching SDG interlinkages from a technology context.

The largest proportion of research on the SDG interlinkages have taken off from a specific goal or used a nexus approach. For example, Fuso Nerini et al. (2018) and Singh et al. (2018) both focused on one specific subject (respectively, energy and water) and took one specific SDG as a starting point by making only SDG 7 Affordable and Clean Energy and SDG 14 Life Below Water central in their analysis. However, when assessing sustainability for a technology, it is important to take a systems approach, meaning that all three dimensions of sustainability—social, economic and environmental—should be assessed, as this triple bottom line perspective determines whether a technology is truly sustainable (Elkington 2004). Tremblay et al. (2020) did include all three dimensions by grouping the SDGs in five pillars (people, planet, prosperity, peace, and partnerships), but did not take interlinkages between goal targets into account. Recently, there has been an increasing amount of papers that have focused on interactions across all (or a large set of) goals (e.g., Anderson et al. 2022; Hernández-Orozco et al. 2022; Warchold et al. 2021; Weitz et al. 2018). To comprehensively assess the links of a new technology toward the SDGs, it is of importance to include all relevant SDGs within the analysis.

## Method

### Research context

This paper studies the interlinkages in the context of the technology PoC microfluidics. This technology was chosen because it is a relatively new technology, which has been widely diffused over the last couple of years, resulting in several concerns that are interesting to connect to the SDGs. Next to that, its wide application areas make it an applicable technology to connect it to the broad focus of the SDGs. In addition, the recent attention during the COVID-19 pandemic given to the sustainability issues related to the technology imposes additional interests of researching this more in depth.

The nowadays most well-known microfluidic point of care test is the rapid antigen COVID-19 test. PoC devices can be used to make diagnoses (and therefore healthcare) more accessible, especially in rural areas (Jani and Peter 2013; O’Kane 2014). This has several positive implications on social sustainability objectives, such as reducing inequality and access to health. PoC testing is laboratory testing, but without needing an actual laboratory as testing is conducted at “the point of care” while providing rapid results (Mejia-Salazar et al. 2020; Nichols 2007). Microfluidics is the name given to the technology for the fabrication of microminiaturized devices containing channels and chambers, with scale dimension in the order of 1 mm or less, to control the flow behavior of small volumes of fluids (Gale et al. 2018). This technique could be used for the lab-on-a-chip technology and could therefore provide rapid diagnostics even in remote areas (near a patient) with limited resources or even in non-existant healthcare settings (Mejia-Salazar et al. 2020). However, the environmental friendliness of PoC devices has been regarded as one of the major challenges, as these devices do not yet allow reuse, which also has a negative effect on the price of the product (Isiksacan et al. 2016; Mejia-Salazar et al. 2020; Trantidou et al. 2018).

### Data collection

To study the interlinkages, an expert panel of relevant stakeholders with interdisciplinary backgrounds was used, which is a widely used method to study SDG interactions, according to Bennich et al. (2020). According to Folke et al. (2016) and Weitz et al. (2018), experts’ perceptions can have strong implications for SDG implementation and an expert panel is therefore an appropriate method to study SDG interlinkages. Previous research showed that especially when studying the target level of the SDGs, using experts’ and stakeholders’ subjective knowledge to characterize the interactions of the SDG targets has been proven to be a good method fit (Bennich et al. 2020; Hernández-Orozco et al. 2022). Bennich et al. (2020) also pointed out that expert and stakeholder knowledge plays a vital role to understand the highly contextual SDG interactions and to bridge the gaps in (and lack to access to) data and literature.

#### SDG interlinkages methodology

**Fig. 1:**
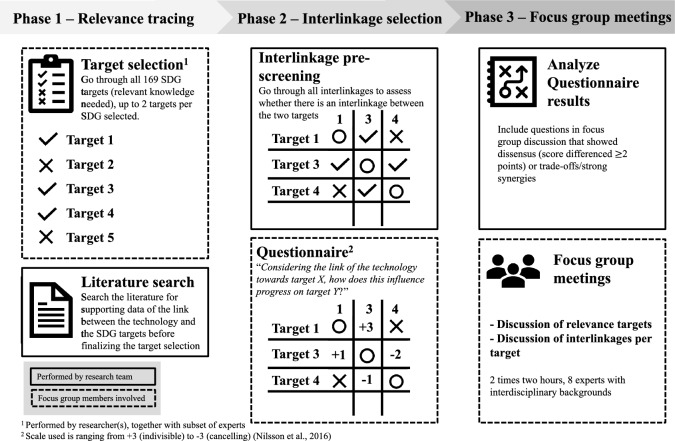
SDG interlinkages methodology phases

For this research, a novel methodology has been developed to study the interactions of the SDG targets in detail. It is based on the approach used by Weitz et al. (2018), but has been adapted to make it fit for studying the context of a technology. Studying interlinkages in a technology context is different compared to policies, as not every SDG will link toward the technology. Data collection took place in three phases: relevance tracing, interlinkage selection and the focus group meetings, as presented in Fig. 1.

This methodology aims to include all relevant SDGs (with up to two targets per SDG) for the technology, and—to keep the analysis manageable—involves several phases to filter the relevant interlinkages to minimize the chances of missing out on interesting ones. Multiple data sources were used (focus groups, existing literature and a questionnaire) and multiple researchers and interdisciplinary experts were included in the research to ensure robustness, i.e., triangulation. The first phase therefore involves selecting the relevant SDG targets that link the technology. During this step, a target selection session with a subset of the experts is organized where the relevant targets (up to two targets per SDG) are selected. Additionally, a literature search is performed to add existing and relevant data to the selected targets for the technology. Here, this resulted in 19 included targets across 12 SDGs. An overview of the selected targets and how PoC microfluidics links toward these targets is listed in Appendix 2. During the second phase, the targets are plotted against each other in a matrix (i.e., interlinkages are formed, 361 in total), and the irrelevant interlinkages are screened out by the research team (the two authors and one additional administrative team member who was also present during the entire process), again to keep it manageable. In case of uncertainty, the interlinkage was not screened out and left in the analysis. When the selection of the targets was finalized, the experts received a preparation document with information about the research, the technology, the SDGs and the selected targets. After that, the experts filled in a questionnaire where they scored the interlinkages (± 100 interlinkages were included). For each interlinkage, the question “Considering the link of the technology toward target X, how does this influence progress on Target Y?”. To answer this, the scale of Nilsson et al. (2016) was used^1^, where the interlinkages were scored from − 3 to + 3. In the third phase, the data of the questionnaire was analyzed and the focus group protocol where the interesting interlinkages were included to discuss in depth was formed. These interesting interlinkages could be when there was dissensus between the experts’ answers (more than two points difference in the answers), when trade-offs were scored (from − 1 to − 3), or when strong synergies (from + 2 to + 3) were scored. Finally, two focus group meetings of 2 h each were performed, which covered the relevance of the SDG targets to the technology and the interlinkages between the targets. The relevance of each selected target was discussed first, after which the discussion moved to the interlinkages the target has with other targets. Within these focus group meetings, 19 targets were included and 28 interlinkages were discussed. The focus group meetings were recorded, fully transcribed and uploaded in NVivo.

#### Selecting the focus group participants

When using focus group research, the results are very dependent on the experts recruited. It is therefore essential that the selection of these experts is thought through carefully. Theoretical sampling was used to select the experts with interdisciplinary backgrounds. Three types of experts were recruited, with knowledge in the following areas: SDGs, application of health technologies (in low- and middle-income settings) and PoC manufacturers or PoC engineers. An overview of the experts can be found in Table 1. These interdisciplinary backgrounds were chosen to ensure knowledge across all SDGs was present. Experts were selected from the researchers’ network, LinkedIn, or via research papers. In total, 15 experts were recruited and screened, resulting in 8 experts selected. The screening consisted of an individual meeting with all participants to gain in-depth insights into the background of the experts, to set the right expectations and to explain the procedure of the participation. The reasons for experts not being selected varied from not meeting the knowledge criteria, scheduling issues and time zone issues. The selected experts came from different countries, mainly in Europe, such as Norway, Belgium, The Netherlands and Spain, but most of them have worked in different countries. The selected experts were fully prepared before participation in the research. The preparation involved providing background reading materials about the technology and the SDG framework.

**Table 1:** Overview of experts and their backgrounds

	Background	Location of research
Expert A	Researcher with extensive experience in the SDG framework	Norway
Expert B	Researcher within assistive health technologies and digital innovations in low- and middle-income countries	Norway + Tanzania
Expert C	Pediatrician, entrepreneur in digital innovation within health and researcher in health technologies	Norway
Expert D	Professor in health systems and global health, specialized in HIV and hepatitis	Spain + United States
Expert E	Researcher in nanotechnology and point of care microfluidics	Norway
Expert F	Engineer working in microfluidic development and bioassay integration for PoC technologies	Belgium
Expert G	Biomedical engineer and innovation manager in biosystems working with PoC microfluidics	Belgium
Expert H	Industrial engineer, researcher in medical devices and innovations for affordable health and health innovation manager in low- and middle-income countries	India + Norway + The Netherlands

### Data analysis

This research has used an abductive approach for coding, as mentioned by Dubois and Gadde (2002). The codes used within the analysis are deducted from and based on the language used in the SDG targets, but also other codes not related to the SDGs inductively emerged. For data structuring, first-order concepts, second-order concepts and themes were established, as shown in Table 2 (Gioia et al. 2013). For data analysis, three phases have been completed: open, axial and selective coding, as proposed by Corbin and Strauss (1990).

*Step 1:* During the first step, open coding was performed, where the direct and indirect links of the technology toward the SDG targets were identified, i.e., “relevance tracing”.

*Step 2:* In the second step, axial coding linked the relevance (direct/indirect links) of the targets to the synergies and trade-offs. In other words, during this step, the relations between the several concepts were identified and it was analyzed from which variables the synergies and trade-offs happened.

*Step 3:* The third and last step of the analysis consisted of selective coding. Here, other factors (e.g., enabling factors) that influence the trade-offs, synergies, direct and indirect links of the framework were analyzed. During this step, the variables were also linked to the selected targets, and, if needed, to additional targets.

**Table 2:** Representative quotes supporting second-order themes

Concepts	Proof quotes
*Theme 1: Direct impacts*	
Direct positive effect on access to basic services	“I think PoC devices can have a good impact on the accessibility to healthcare by simplifying the whole procedure and making it more low cost so that people don’t need to travel that far anymore, especially in low- and middle-income countries.”—Expert F
Direct positive effect on fight communicable diseases	“It has clearly showed that over the past ten years, the incidents and prevalence have been reduced significantly due to the introduction of these tests”—Expert G
Direct negative effect on sustainable material use	“Although the strip itself is paper, which you could argue is more disposable and less impactful on the environment, everything around it is plastic”—Expert G
Direct negative effect on recyclability and waste generation	“Microfluidic tests are being burnt after usage because they are seen as medical- and biocontaminated-devices. In that way it will also lead to pollution of air.”—Expert D
*Theme 2: Synergies in socially related targets*	
Synergies to reducing poverty and income inequalities	“If the PoC tests diagnose early and if the tests are for free, it could reduce inequalities. People can continue work and can get a job.”—Expert B
Synergies to gender equality	“It gives access to pregnancy tests, but also to diagnosis of sexually transmitted infections. Here, where women generally globally have less access to healthcare and maybe challenging access around these particular issues, these tests are a real game changer.”—Expert D
*Theme 3: The (enabling) socio-economic factors*	
The enabling role of Universal Health coverage	“I think here, again, the link with universal health coverage is accessibility to healthcare and to diagnosis. And accessibility can be improved by these point of care systems.”—Expert A “The reason people don’t access healthcare, is because they have greater issues to worry about. Achieving universal health coverage could change this”—Expert H “You just need technology like Point of Care devices for Universal Health Coverage to exist.”—Expert D
The role of price for accessibility	“The price of these tests is key. If you want to develop it in low- and middle-income countries, that’s where actually some of these tests are failing at this point.”—Expert G
The role of conveniency of the product	“PoC diagnostics are complete game changers because you can reach marginalized populations, by going out into the field and test with those kinds of tests.”—Expert D
PoC and job and skill creation	“Most often diagnostic devices are quite high-tech and it will create jobs within manufacturing and create economic productivity.”—Expert F “When microfluidics is used more, other interventions, tools and devices will be used less. How do we know this will create positive economic growth? It might be compensated through a loss in other areas.”—Expert D “Maybe the active working period of people can also be extended by using PoC, because they don’t need to travel to hospitals anymore to do checkups. Then they don’t need to take day off to go to the hospital. Through more personalized medicine, people often stay longer on the same treatment without adverse effects. This way, they don’t relapse, which also leads to an extended period of active working time of people.”—Expert F
*Theme 4: Trade-offs in environmentally related targets*	
The trade-off between better access to health and green sustainability	“I think an improved exposure to good healthcare always will go along with a negative effect on efficient use of natural resources, because it is all disposable material.”—Expert F “Do you really expect those countries lagging behind to take on all these missions at the same time? If you want to have better access, do you have the resources to take into consideration all the sustainable goals at the same time? It’s just a lot to ask. If you have to prioritize, would you have better access to healthcare, or do you want to have natural resources being spent in a certain way? I think it’s just very difficult to expect that kind of development to happen at the same time.”—Expert C
The influence of recyclability and sustainable material use on price	“Having environmental objectives in mind would increase costs and make things more difficult, compared to just rolling something out to increase access. Plastic is a fantastic product when it comes to transportation and making products safe and storage friendly.”—Expert C

## Findings

### Direct impacts of PoC microfluidics on SDG targets

**Fig. 2:**
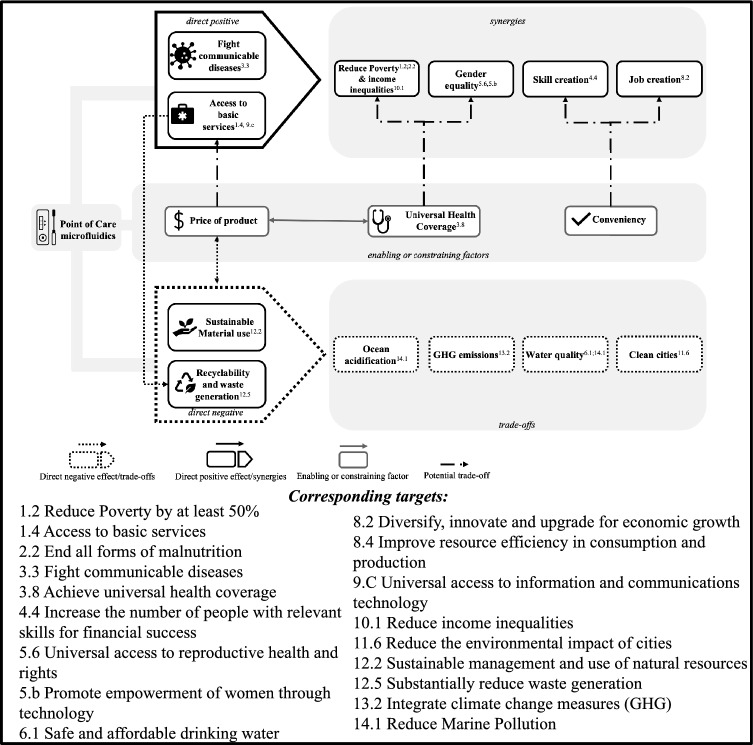
The linkages and interlinkages of point of care microfluidics toward SDG targets

The examination of SDG interlinkages in the context of PoC microfluidics provided insights into how this technology strongly links with certain targets and how these targets in turn interlink with other targets, as shown in Fig. 2.

The technology has a direct positive impact on the social targets 1.4 *Access to basic services* and 3.3 *Fight communicable diseases*. PoC microfluidic testing enables accessibility to health care, because it simplifies the procedure and therefore also brings the costs down. When the technology (and thus access to diagnostics) becomes better available, better insights into the incidence and spread of communicable diseases is possible (linking to target 3.3). The COVID-19 pandemic is a very good example of how these microfluidic tests play a very important role in fighting a worldwide pandemic. But for low-resource populations where other communicable diseases, such as HIV and hepatitis B, are causing millions of deaths yearly, these diagnostic tests can reach people which are otherwise not diagnosed:

“For HIV and hepatitis B, PoC diagnostics are complete game changes because you can reach marginalized populations”—Expert D

In this case, especially the fact that these devices are completely independent of current existing infrastructure in the local community is a big asset. Because of this, you can reach marginalized populations that you could not have reached if you would have been dependent on an existing infrastructure to diagnose these diseases. By all means, it should be noted that there should be an infrastructure in place to ensure a proper follow-up after a positive test, i.e., referral to a centralized facility to cure and care for the patient.

Another finding that emerged out of the data was the direct negative impact the technology has on the environmentally related targets (8.4, 11.6, 12.2, 12.4 and 12.5) on *sustainable material use* and *recyclability and waste generation*, also having negative effects on water quality (targets 6.1 *Safe and affordable drinking water* and 14.1 *Reduce marine pollution*). Because of the high amounts of plastics associated with the devices and the fact that after use it consists of contaminated materials, the use of these devices creates a lot of waste. This leads to several environmental concerns:

“Increased plastic usage will always have a bad influence on marine pollution and pollution in general”—Expert F

From these direct impacts, several synergies and trade-offs have been identified. Throughout the further explanation of findings, the emerging concepts and its underlying relationships will be explained in more detail. Further data for each emergent theme is provided in Table 2.

### The social synergies associated with PoC microfluidics

PoC microfluidic devices can play a major role in making healthcare more accessible—especially in marginalized communities. As these devices make healthcare more accessible, *access to basic services* will increase (SDG target 1.4). One important point that should be considered when looking at the impact PoC microfluidics can have on basic services is that these diagnostic devices are part of an ecosystem, meaning that there should be a system in place where a proper follow-up after a positive test is possible:

“There needs to be a system available so that you can follow up. Sometimes you get a positive test and what do you do if there’s no treatment or facilities available. Otherwise, the impact can even be negative, because you are just diagnosing something, and you don’t know what to do and you are just causing anxiety.”—Expert C

As target 1.4 *Access to basic services* is part of SDG 1 *No Poverty*, progress on this target creates synergies toward other poverty related targets, such as targets 1.2 *Reduce poverty* and 10.1 *Reduce income inequalities*. When access to health is ensured, people are healthier (linking to the same synergy running from target 3.3 *Fight communicable diseases*) and are able to provide an income for themselves and their families.

Access to these PoC devices and fighting communicable diseases also creates synergies toward gender equality. Globally, women have less access to healthcare and diagnostics than men and simplifying access to health and diagnostic tools has become increasingly more important to fight these gender inequalities. These devices can not only perform pregnancy tests, but also play a large role in diagnosing sexually transmitted diseases. Once more, the independency of infrastructure facilitates how this technology can play a crucial part for women to gain control over their own health:

“Women might have to use different types of technologies to get access to abortions, because of laws and regulations. That would mean there is a new wave of creative, innovative ways to ensure women get a hold of what they need to gain empowerment over their own bodies.”—Expert A

### The (enabling) socio-economic factors related to PoC microfluidics

One fundamental condition to create the social synergies is the price of the product and who in turn pays this price (hence the central position of “price of the product” in Fig. 2):

“The cost of these tests is key. If you want to develop it in low- and middle-income countries, that’s where actually some of these tests are failing at this point.”—Expert G

However, it is ambiguous whether PoC microfluidics actually brings the costs of healthcare down. In the case of hepatitis testing in Spain, it was mentioned that it actually increases the costs of healthcare while greatly enhancing access. This links directly toward target 3.8 *Achieving Universal Health Coverage*, which should ensure that people have access to essential healthcare (hence the enabling character of the PoC microfluidics technology) without being financially sacrificed (hence the enabling character of the price of the product). Instead, if these devices are not covered by universal health coverage (or when the price of the product becomes too high), it could negatively affect reducing poverty and reducing income inequalities. This is the case when marginalized communities (such as low- and middle-income countries) do not have the means to pay for the device—whereas higher-income communities do—and thus increasing poverty and inequalities in income and access to health:

“If people have to pay for the point of care product, if it’s not covered with a universal healthcare coverage, for example, that could negatively affect reducing income inequalities.”—Expert B

The effect of the deployment of PoC microfluidics on job and skill creation was identified as ambiguous. On the one hand, it was mentioned that when these devices are being deployed on a large scale that this will always create additional jobs and possibly also skills. On the other hand, when looking at the use of PoC microfluidics in urban areas, this effect will be minimal or even negative as it might take jobs away from the laboratories and other health facilities. This relates to the fact that the use of PoC microfluidics does not need any additional infrastructure and in some cases does not even need any additional skills. This means that the conveniency of the product creates enormous opportunities for large-scale deployment, but can also counteract skill and job creation. On the other hand, in rural areas (where no, or limited healthcare infrastructure is present) PoC microfluidic devices can create jobs in the local communities, because patients do not need to go to centralized facilities to get diagnostic services. In turn, these devices can also enable local nurses or doctors to develop additional diagnostic skills. This means that in rural areas, the deployment of these devices and the presence of diagnostic services can bring an entire new life to the local communities.

### The environmental trade-offs associated with PoC microfluidics

Since the COVID-19 pandemic and the enormous push it gave to the deployment of microfluidic tests, the environmental sustainability issue concerning these tests has been put higher on the agenda. Especially, the waste these tests (along with other medical disposables such as mouth masks) produce since the COVID19 outbreak has been identified as one of the greatest concerns:

“As we push to improve healthcare, there will be a toll on natural resources. We will work to make technologies better, but I mean just looking at the waste from the rapid antigen tests is a clear example of what happens to the environment when you’re hoping to improve human health.”—Expert D

The environmental issues concerned with microfluidic tests link toward several SDG targets. The material use (i.e., plastics), waste generation and lack of recyclability of these tests negatively influences targets 11.6 *Reduce the environmental impact of cities*, 12.2 *Sustainable management and use of natural resource* and 12.5 *Substantially reduce waste generation*. Recycling these tests is a very challenging task, even if it was comprised of better recyclable materials. First, these tests carry biological waste after they have been used and therefore regulations ensure that these tests must be burnt after use. Second, the costs of recycling are higher than producing a new product:

“I think the business problem here is that it is easier to produce new product than recycling it. From the industry point of view, it is not exciting for me to really invest in recycling. I rather produce new and continue the business.”—Expert H

This means, if progress would be made on using more sustainable materials (e.g., recyclables), it would currently create a trade-off toward the accessibility of the product as the price of the product would rise:

“If you make progress on more sustainable products then some of these devices may even be more expensive, even though they may increase access to healthcare.”—Expert D

## Discussion

### SDG interlinkages: identified trade-offs and synergies

As illustrated in Fig. 2, the findings suggest that in the context of PoC microfluidics, the interlinkages originate from several direct links of the technology with the SDG targets. Positive direct links occurred between social- and economic-related SDGs (access to basic services and fighting communicable diseases), which in turn creates several synergies on what Lehoux et al. (2018) call “social determinants of health”. The social determinants of health identified in this study include reducing poverty (SDG target 1.4), reducing inequalities (SDG target 10.1), enhancing gender equality (SDG 5.6 and 5.b), skill creation and job creation (SDG 4.4 and 8.2). The conveniency factor of this technology has shown to be a great asset to deployment on a larger scale. Previous research already mentioned how this can positively affect reducing inequalities, as these services are not labor intensive, do not require specialized health personnel and do not necessarily need to be connected to urban centers (Hurst 2000). Although this can also create trade-offs for skill creation, deploying PoC technologies means that also rural- and low-resource settings can have access to basic primary care which significantly reduces inequalities in these regions (Charlton et al. 2015). Previous studies have emphasized the reciprocal relation between access to healthcare and poverty and inequality (Peters et al. 2008): when healthcare is needed, but cannot be financed and is delayed or even not obtained, health conditions worsen. In turn, this leads to loss of income and higher healthcare costs, both contributing to an increased rate of poverty (Narayan et al. 2000; Smith 1999). The results have shown that this can also work the other way around: when healthcare is (earlier) obtained, income can be generated, and inequalities are reduced. However, one crucial part of this argument (as also mentioned in the argument of Narayan et al. 2000; Smith 1999) is that the user of the technology should not face financial burden for these synergies to occur. The findings have stressed the importance of universal health coverage to enable these synergies.

PoC microfluidics also creates negative links and trade-offs toward SDG targets. Direct negative links occur on environmental targets related to recycling, waste management and the sustainable use of natural resources. Especially the material use, i.e., plastics, plays an important role in the negative impacts and the trade-offs associated with this. Ji et al. (2022) estimated that the emissions of COVID-19 testing in 2021 (4.21 billion tests in the 122 countries in their analysis) was 2.28 million tons of CO_2_, which constituted 1.7% of the total carbon footprint of Japanese healthcare services or 10.7 times the annual GHG emission of hysterectomies in the USA. However, the exact negative impacts of the use of plastic depends on how it is produced and how the waste is managed. In fact, the use of plastic as a material has several advantages, such as durability, flexibility and the price (Heidbreder et al. 2019). The worldwide plastics problem can be seen as a so-called “wicked problem”: problems that are ill-formulated, with confusing information, with many actors that have conflicting values and where the proposed “solution” often turns out to be worse than the symptoms of the problem (Lönngren and Van Poeck 2021). Previous studies already underlined how technical solutions to plastic are often undermined by psychological effects such as increased use (i.e., rebound effects) or increased littering of biodegradable products (Haider et al. 2019; Hertwich 2005). It is therefore crucial that solutions, apart from policy-oriented solutions, also focus on human behavior in an interdisciplinary manner to limit the (unnecessary) use (i.e., reduce) and recycling of plastic (Heidbreder et al. 2019). However, the main problem with PoC microfluidics is that it contains contaminated materials and chemical waste, which cannot be recycled and need to be incinerated (leading to high amounts of GHG emissions) (Ongaro et al. 2022). According to Ji et al. (2022), the total GHG emissions of a COVID-19 nucleic acid test (NAT) are 612.90 g CO_2_ and the waste treatment contributes to 71.3% of these GHG emissions. They estimated that improved waste treatment (using movable microwave incineration equipment) can reduce 16.3% of air pollutants, 18.8% of heavy metals and 61.7% of water pollutants. Other solutions Ji et al. (2022) proposed that can bring the environmental footprint of COVID-19 NAT down are electric vehicle transportation (reduction of 17.1% in air pollutants, 24.3% in heavy metals and 7.7% in water pollutants) and sustainable material substitution (reduction of 16% of GHG emissions, 25% heavy metals, 23.1% of air pollutants and 9.3% of water pollutants).

### SDG interlinkages and responsible innovation dimensions and responsibilities

**Fig. 3:**
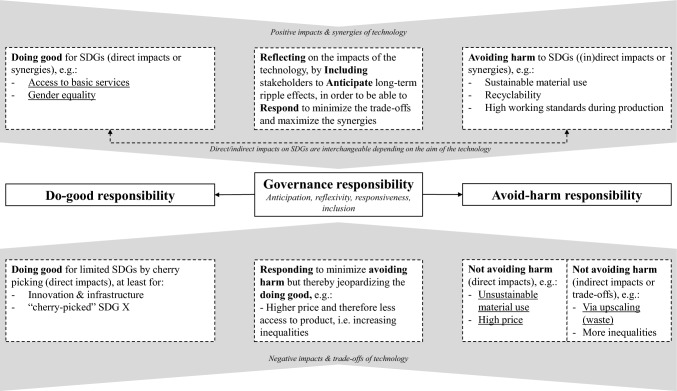
How SDG interlinkages inform the responsibilities and dimensions of the RI framework

As mentioned before, the synergies and trade-offs identified in this study are context specific and therefore not easily generalizable in a wider context than PoC microfluidics. However, the results of this study can be used to link the SDG interlinkages field toward the responsible innovation field. In the case of PoC microfluidics (as underlined in Fig. 3), this means that the positive impacts the technology has on access to basic services and gender equality reflects the do-good responsibility of RI. At the same time, the technology was not avoiding harm as it uses unsustainable materials and creates high amounts of waste. Identifying the (inter)linkages of a technology toward the SDG targets helps to assess the responsibility of the technology in several ways (i.e., direct/indirect, positive/negative impacts, synergies and trade-offs). The other way around, the dimensions (anticipation, reflexivity, responsiveness and inclusion) and responsibilities (governance, do good and avoid harm) included in the RI framework also gives direction on how technologies could become compliant with the SDGs.

#### Governance responsibility

As mentioned by Voegtlin and Scherer (2017), the governance responsibility plays a crucial part in responsible innovations as it facilitates the do-good and avoid-harm responsibilities. Firstly, this relates to the role of policy makers and regulators. For example, in the case of plastic, there are two main types of policy instruments that are aimed at reducing plastic use: bans and economic policy instruments (e.g., fees, levies or taxes) (Heidbreder et al. 2019). These policies should facilitate innovators to reduce the use of plastic in their product and thus should enhance avoiding harm and/or doing good.

With the introduction of the SDGs, direction is given to compliance with sustainability objectives and which policies and regulations are needed to enforce that. However, previous research also warned that because the SDGs cover a broad range of topics, the SDGs may also be used to show compliance with goals that they comply with anyway, i.e., cherry-picking “friendly goals”, increasing the danger of greenwashing (Nylund et al. 2022). Therefore, the notion of “governance” should be interpreted wider than only the role of policy makers, as it should no longer be something that governments do exclusively, but now also should involve non-state actors working with, or even governing without, governments (Florini and Pauli 2018). The four dimensions (anticipation, reflexivity, responsiveness and inclusion) related to the RI framework (Stilgoe et al. 2013), should therefore be used to execute this governance responsibility. Reflexivity is described as “holding a mirror up to one’s actions, commitments and assumptions” (Stilgoe et al. 2013, p. 1571) and can be achieved through processes and activities such as ethical technology assessment. The SDG interlinkages methodology used within this paper can be seen as a proper tool to do this assessment. This assessment also includes anticipation, as it assesses the future impacts and implications of the technology (Jakku et al. 2022) by using the 2030 Agenda which the SDGs are part of. As emphasized by previous scholars, reflection and anticipation rely on the inclusion of relevant stakeholders to discuss the consequences of the technology, which increases the diversity of inputs and encourages transformative mutual learning (Bäckstrand 2006; Jakku et al. 2022; Thompson et al. 2017). By using focus group research with experts with interdisciplinary backgrounds, this SDG interlinkages method aims to start this dialog among different stakeholders. So, by analyzing the SDG interlinkages, one can *reflect* on the impacts of the technology, by *including* the relevant stakeholders with interdisciplinary backgrounds, to *anticipate* on the long-term ripple effects to be able to *respond* to minimize the trade-offs and minimize the synergies. In turn, this should ensure that the technology is doing good while avoiding harm.

#### Positive impacts and synergies of the technology

The results indicated that technologies that are “doing good” can have multiple positive direct effects on the SDGs. In the case of PoC microfluidics, this was reflected in the direct impacts on the SDGs of access to basic services and fight communicable diseases. From the effects of directly “doing good” with the technology, this could in turn create several synergies (e.g., toward gender equality) and hence even “doing more good”.

As previously mentioned, for technologies and innovations to be truly responsible, they should at the same time also avoid harm (Voegtlin and Scherer 2017), which is reflected via the indirect impacts of the technology on the SDGs. If PoC microfluidics were truly responsible, this would mean that the technology is produced from sustainable materials, and/or recycling strategies are in place. Whether the impacts of the technology on the SDGs account to avoiding harm or doing good depends on the main aim of the technology: in the case of socio-innovations the social SDGs will be directly impacted (i.e., do good) and the environmental SDGs will be indirectly impacted (i.e., avoid harm), whereas in the case of eco-innovations the environmental SDGs will reflect the do-good responsibility.

#### Negative impacts and trade-offs

The deployment of innovations and technology will usually have a direct impact on at least a limited number of SDGs, albeit only on targets of SDG 9 Industry Innovation and Infrastructure (e.g., target 9.5 *Enhance research and upgrade industrial technologies*). In that way, even when a technology does not qualify as a responsible innovation, it will be doing good in some way. As previously mentioned, technologies can also directly impact “cherry-picked” SDGs, as these goals are in itself already very closely related to the outcomes of the technology. Previous scholars emphasized the danger of cherry-picking SDGs, as this could be used “to camouflage business-as-usual by disguising it using SDG-related sustainability rhetoric” (Bebbington and Unerman 2018, p. 10). Buhmann et al. (2018) stated that from the neo-institutional perspective, organizations engage with the SDGs primarily to respond to institutional pressures, rather than to significantly improve sustainability practices and performance, also known as greenwashing. In the case of compliance with the SDGs and other UN initiatives, a specific term was even created: bluewashing (with more emphasis on the economic and social factors), referring to the blue UN flag (Berliner and Prakash 2015). From a technology point of view, blue- and/or greenwashing happens when the positive sustainability impacts of a technology are exaggerated and/or overemphasized. The method used within this paper to study SDG interlinkages of a technology could expose bluewashing, as it comprehensively investigates the links toward all the SDGs.

In the case of PoC microfluidics, it became clear that the technology was not avoiding harm, relating to the direct negative impacts on the environmentally oriented SDG targets. In addition, technologies can also not avoid harm indirectly and via trade-offs: the upscaling of the technology during the COVID-19 pandemic created an amount of waste that was not foreseen. The results indicated that when responding to the trade-offs to minimize avoiding harm, a close eye should be kept on the doing good responsibility, as this might create additional trade-offs. This can be the case when other materials would be used, resulting in a higher price which will limit the wide accessibility of the product, jeopardizing all the associated synergies. This relates to what Stilgoe et al. (2013) mentioned as the “societal embedding of technologies”, which requires a process of alignment. Technologies are part of a sociotechnical system, where synergies and trade-offs between factors occur and where actors and interests are arranged such that they are dependent on one another. This means that responding to new insights because of reflexivity, anticipation and inclusion (and thus “changing” this sociotechnical system) requires understanding the alignment of these factors. Stilgoe et al. (2013) proposes to respond in a way that Fisher et al. (2006) describes as three types of decision-making: de facto, reflexive and deliberate. This should ensure that the assumptions are more explicit and decisions more deliberate.

### Implications

#### Implications for the SDG interlinkages research stream

This study responds to the recent calls to emphasize the indivisible nature of the SDG framework instead of treating it in separate silos (Bennich et al. 2020; Imaz and Sheinbaum 2017) and the role technology and innovations can play to impact the SDGs (Scheyvens et al. 2016). One implication is that the identified synergies and trade-offs emerge from a handful of strongly directly related targets toward the technology, instead of synergies and trade-offs emerging scattered throughout the entire SDG framework. The latter has been the case when SDG interlinkages have been researched from a policy perspective, for example, in the papers of Castor et al. (2020) and Weitz et al. (2018). This implies that from a technology perspective, it is very important to first map the direct and indirect links toward the SDGs in detail, before heading toward the interlinked effects.

One of the critiques of previous scholars on the SDGs is that the SDG framework does not allow for prioritization of one of the three dimensions (economic, environmental and social). According to Griggs et al. (2013) and Le Blanc (2015), the SDG framework does not have coherence of the nature of the “socio-economic engine” and this lack of coherence allows for conflicts among and within goals. This view is in line with the identified trade-offs within this paper. Nonetheless, using the SDG interlinkages framework to identify how these conflicts within and between goals can occur enables priority setting within the SDG framework and thus can accelerate goal achievement.

#### Implications for RI

Using the SDG (interlinkages) framework has proven to be a fruitful framework to study whether a technology is a responsible innovation, as it addresses the ethical, economic, social and environmental principles needed for a responsible innovation (Khayesi 2021). Analyzing SDG interlinkages builds on the dimensions (anticipation, reflexivity, inclusion and responsiveness) of RI and thus provides a comprehensive and systematic way to analyze the level of “responsibility” of a technology, by taking the long-term ripple effects into account. This research adds the long-term focus—as being part of the anticipation dimension—to the analysis of a responsible innovation as it reflects on the long-term impacts of the technology by including relevant stakeholders. In addition, it makes it possible to analyze whether a technology relates to the “do-good” responsibility, but more importantly if it also relates to the “avoid-harm” responsibility.

#### Practical and policy implications

As this paper builds on the policy related and practical SDG framework, this work has several practical and policy implications. First, this research has shown that researchers and manufacturers should focus on ways to make the technology more environmentally sustainable without putting the accessibility of the product at risk. Here, it is crucial that the materials of the device have characteristics that ensure that less waste is created after use. Recently, an increasing amount of scholars have been researching solutions for the sustainability issue of PoC technologies. For example, Ongaro et al. (2022) proposed several solutions along the life cycle of PoC microfluidic devices, such as renewable raw materials, local production, use of sustainable reagents, use of mobile health solutions and on-site waste facilities. However, the manufacturing of plastic-alternative medical products has been shown to be more complicated than the substitution of plastic in other sectors (Celis et al. 2021). Celis et al. (2021) proposed reduction in taxes, subsidies and incentives in R&D for development of low or no toxicity compounds to support these advances without jeopardizing the low cost of the product. Nonetheless, it is also crucial that policy instruments focus on the behavior of how people deal with the product after use.

The use of technology to reduce gender and income inequalities (and to achieve other socially related goals) stands or falls with the availability of the technology for all. As the main intention of the SDGs is to *leave no one behind*, technologies can only ensure progress on these SDG targets if they are available for all. If not, technological innovations should be doubted as a symbol of progress and the opposite might be true, meaning that technological innovations might even increase inequalities (Weiss and Eikemo 2017). This directly relates to the crucial role universal health coverage plays. It is only with universal health coverage that low- and middle-income countries can reap the rewards from (health) technologies in the same way as high-income countries can.

## Conclusion

The main goal of this paper was to investigate SDG target interlinkages in the context of a technology. Technologies are seen as pivotal to achieve the SDGs and the long-term interlinked effects of technologies on the SDGs can undermine or accelerate the progress achieved in the short term. This paper demonstrated that studying SDG interlinkages from a technology perspective can inform the RI research stream in several ways. First, it shows that SDG interlinkages can be used to execute the governance responsibility through the RI dimensions anticipation, reflection, inclusion and responsiveness. Second, it provides insights into if and how a technology does good or avoids harm and assigns this to the SDG targets. Therefore, using the SDG target interlinkages methodology offers a tool to assess the responsiveness of a technology in depth.

To our knowledge, this is the first study to research SDG targets interlinkages in the context of a technology and a novel methodology has been developed to do so. Therefore, a number of limitations should be considered. First, even though this method aims to include as many SDG targets (and corresponding interlinkages) as possible, it may not be able to capture all the positive and negative impacts the technology has on the SDGs. This study only included eight experts and performed three focus group sessions, which is a limited amount of respondents and data collected. However, this study was of an exploratory nature to understand how analyzing SDG interlinkages works from a technology perspective. In other words, in this study the aim was to look broader and connect this topic with responsible innovation, instead of solely focusing on the empirics of PoC microfluidics. Future research (qualitative and quantitative) is needed to validate the results and gain a deeper understanding on SDG interlinkages for the chosen research context. Second, even though the experts that were selected have international research experience and worked in low- and middle-income settings, it should be noted that all experts were based in Europe. Future studies could therefore include experts from other regions in the world to see if other synergies or trade-offs will be identified and whether the results are generalizable across different world regions. Third, the current study has only examined one technology, and more technologies are needed to validate the methodology and the connections of the SDG interlinkages to the RI framework. Future research is needed that uses this methodology for more technologies with different characteristics, for example, more complex technologies with large upfront investments that are deployed over a long period of time, such as renewable energy technologies or other novel environmental technologies. Finally, assessing SDG interlinkages shows how the current design or application of the technology links toward SDG targets, but does not involve responsiveness to make the technology more responsible in relation to the SDGs. To this extent, this paper paves the way for future studies that focus on the solutions needed to respond to the identified trade-offs and synergies in this study.

## Appendix 1: SDG interlinkages table

See Table 3.

**Table 3:** Overview of SDG interlinkages research methods and contexts researched

Method used	SDG point of entry	Level	Context	Sources
*Quantitative methods used*				
Correlation analysis	All goals	Target level	Multiple countries	Warchold et al. (2021) and Pradhan et al. (2017)
Network analysis	All goals	Target level	Multiple countries	Lusseau and Mancini (2019)
Correlation analysis and expert knowledge	All goals	Target level	Multiple countries	Anderson et al. (2022)
Network analysis	Multiple goals, divided in three systems	Target level	Multiple countries in multiple regions	Swain and Ranganathan (2021)
Indicator based assessment	Multiple goals	Target level	Single region	Allen et al. (2017)
Integrated assessment model	Set of goals	Goal level	Single country	Hutton et al. (2018)
Multiple factor analysis	Multiple goals	Target level	Multiple provinces in a single country	Zhang et al. (2022)
*Mixed methods* + *qualitative methods used*				
Literature review + correlation analysis	Multiple goals covering several broad areas	Target level	River basin of multiple countries	Zhou et al. (2022)
Expert assessment + network analysis	All goals	Target level	Single country	Weitz et al. (2018) and Hernández-Orozco et al. (2022)
Expert questionnaire + correlation analysis	All goals	Goal level	Multiple countries in multiple regions	Yang et al. (2020)
Literature review + questionnaire	From SDG 7 to all other goals	Target level	Energy projects and/or systems	Castor et al. (2020) and Fuso Nerini et al. (2018)
Expert assessment	From SDG 14 to all other goals	Target level	Oceans	Singh et al. (2018)
Questionnaire + correlation analysis + linear regression	All goals within five pillars	Target level	Pillar (people, planet, prosperity, peace and partnership) interactions	Tremblay et al. (2020)

## Appendix 2: Target selection

See Table 4.

**Table 4:** Target selection and rationales

Target	Rationale
1.2 Reduce poverty by at least 50%	Lack of access to health is an important reason people fall below the poverty line. These marginalized communities are reached with PoC tests. A total of 39 million people fall below the poverty line in India every year alone as a result of health-related expenses (Kumar et al. 2016)
1.4 Access to basic services	PoC microfluidics facilitates access to basic health services. Providing primary care in underresourced settings can be performed by PoC technologies which are compatible with these extreme environments (Smith et al. 2016)
2.2 End all forms of malnutrition	PoC tests can be used to diagnose malnutrition. Also, it can test the quality of food (Li et al. 2016) Several diseases are found to link toward malnutrition. For example, children with malaria are found to have poorer nutritional status (Sachs and Malaney 2002)
3.3 Fight communicable diseases	PoC facilitates access to diagnostic services for communicable diseases. It has been reported that lack of proper diagnosis and access to health care are the reasons for more than 95% of deaths due to infectious diseases (Yager et al. 2008). Lack of proper diagnosis also increases the spread of diseases, in the case of HIV it was estimated 20% does not know they have HIV (Soares et al. 2020; Yang et al. 2022) There are concerns that the contaminated medical waste from PoC microfluidics can result in reverse zoonoses (infectious diseases spillover to animal populations, like the 2022 monkey pox pandemic) (Messenger et al. 2014; Ongaro et al. 2022)
3.8 Achieve universal health coverage	PoC tests can be seen as a means to provide UHC, as it provides a low-cost diagnostic solution. Vice versa, UHC is needed to provide PoC without marginalized populations facing a financial burden to access it. Currently, PoC microfluidic testing devices are already an essential component of disease control programs, as it is an affordable option (Agustina et al. 2019; World Health Organization 2019)
4.4 Increase the number of people with relevant skills for financial success	PoC technologies provide employment and training to local health professionals. Currently, most health professionals tend to concentrate on the cities. Agustina et al. (2019) found that in Indonesia less than 10% practise in rural communities, whereas these comprise 45% of the population. The test itself does not require many additional skills, but most skills will be needed by the interpretation of the test and an understanding of how the results inform therapeutic decisions (Haga 2016) Addresses health concerns affecting participation in education. In Kenya it was found that school students miss 11% of school days because of malaria. Absenteeism reportedly increases failure and dropout rates (Sachs and Malaney 2002)
5.6 Universal access to reproductive health and rights	Women generally have less access to healthcare and also challenging access around these issues, particularly STI. PoC gives access to pregnancy tests and diagnosis to STIs. Most of these STIs are easily curable, but are also asymptomatic, and many individuals are not diagnosed in a timely manner, leading to long-term sequelae, such as infertility (Newman et al. 2015)
5.b Promote empowerment of women through technology	Gives women access to enabling technology within health
6.1 Safe and affordable drinking water	PoC microfluidics can detect potential bacteria or contaminants within the water, to assess if the water is safe
	The (contaminated) waste of PoC tests in LMICs ends up in landfills or municipal water supplies. This increases the risk that people will come into contact with the hazardous reagents this waste contains, such as the cyanide derivatives used in PCR cartridges (Ongaro et al. 2022)
7.2 Increase global percentage of renewable energy	Most (and ideally) PoC devices can provide results even in circumstances where the electricity grid is not well established (Liu and Choi 2017). Many paper-based (e.g., pregnancy test) do not need any electricity to be used, but more complicated PoC systems do require energy. This onboard energy source, in most cases solar, is crucial for the deployment of the technology. The new generation of power sources needs requirements such as high power density, low cost and disposability with minimal environmental impact (Esquivel et al. 2014)
8.2 Diversify, innovate and upgrade for economic growth	Epidemics have negative effects on economic growth. For example, Liberia saw GDP growth decline 8 percentage points because of the Ebola outbreak from 2013 to 2014 (Bloom and Cadarette 2019). PoC tests can prevent (further) outbreaks of epidemics PoC tests form new innovations/technological upgrading that can fit into the current health infrastructure while increasing productivity (Laksanasopin et al. 2015; Smith et al. 2016)
8.4 Improve resource efficiency in consumption and production	The projections for the global PoC testing market are to grow to $72B by 2024 from $43.3B in 2022, at an annual growth rate of 10% (Ongaro et al. 2022). However, this economic growth is currently not decoupled from the environmental degradation this industry is associated with
9.C Universal access to information and communications technology	The emerging field of paper-based microfluidics combined with smart phone-based technologies enlarges the need for access to ICT (Smith et al. 2016). In addition, with the implementation of electronic medical records, there is a larger need to communicate test results to these records (Kim and Lewandrowski 2009). With the wider deployment of PoC devices, there is also more need for storing results in an efficient manner to identify trends and to integrate other data, particularly for frequently performed tests (Haga 2016)
10.1 Reduce income inequalities	Diseases like malaria have strong relations toward poverty and income inequality, as low- and middle-income countries bear a disproportionate burden (Bloom and Cadarette 2019; Sachs and Malaney 2002). Access to PoC microfluidics can reduce these inequalities. UHC fights inequalities as health expenditure decreases has been estimated (Agustina et al. 2019)
11.6 Reduce the environmental impact of cities	Especially in LMICs (and to a lower extent also in developed countries), access to PoC tests will place significant additional pressure on waste management systems, especially in cities (Ongaro et al. 2022)
12.2 Sustainable management and use of natural resources	Most single-use PoC tests are made from plastic materials using non-renewable resources. Ongaro et al. (2022) found that 55% of published devices are made of polydimethylsiloxane (PDMS), 12% of silicon and glass, 20% of thermoplastic materials, and 13% of paper
12.5 Substantially reduce waste generation	Over the last 15 years, It has been estimated that about 924,000 L of effluent chemical waste have been generated for HIV testing alone (Ongaro et al. 2022). Most waste of PoC tests are infectious, which should be collected and treated separately. Infectious waste is most often incinerated, increasing the GHG emissions. In LMICs the procedures for treating infectious waste are often lacking, resulting in burning them in open pits and thus increasing the GHG emissions even more as it emits toxic pollutants (e.g., dioxins and furans) (Chartier 2014). In addition, Ji et al. (2022) estimated that a COVID-19 nucleic acid test produces 612.90 g GHG emissions and waste treatment contributes 71.3% to this amount. Waste treatment is also identified as the main source of the other pollutants associated with the tests, contributing to 79% of water pollutants, 62.8% of air pollutants, and 56.0% of heavy metals
14.1 Reduce marine pollution	GHG emissions pollute the oceans as oceans take up GHG from the air. According to Ji et al. (2022), a COVID NAT test produces 3.3 g of air pollutants and 0.88 g of water pollutants

## Data Availability

The participants of this study did not give written consent for their data to be shared publicly, so due to the sensitive nature of the research supporting data is not available.
